# Experimental Investigation of the Effects of ‘*Nigella sativa*’ and ‘*Vitis vinifera*’ on Intraoral Wound Healing

**DOI:** 10.3290/j.ohpd.c_2793

**Published:** 2026-08-26

**Authors:** İsmail Tekbaş, Metin Çalişir, Ebru Annaç, İbrahim Bozgeyik, Ziynet Çinar

**Affiliations:** a İsmail Tekbaş Doctor, Department of Periodontics, Faculty of Dentistry, Adıyaman University, Adıyaman, Turkey. Creating a model in rats, collecting samples, taking blood from the heart, isolating organs and tissues, monitoring experiments, interpreting the results and writing down data analyses.; b Metin Çalişir Professor, Department of Periodontics, Faculty of Dentistry, Adıyaman University, Adıyaman, Turkey. Monitoring experiments, interpreting results, and analysing data.; c Ebru Annaç Associate Professor, Department of Histology and Embryology, Faculty of Medicine, Adıyaman University, Adıyaman, Turkey. Histopathological examinations and interpretation of results.; d İbrahim Bozgeyik Associate Professor, Department of Medical Biology, Faculty of Medicine, Adıyaman University, Adıyaman, Turkey. Biochemical examinations, interpretation of results.; e Ziynet Çinar Assistant Professor, Department of Biostatistics, Faculty of Medicine, Sivas Cumhuriyet University, Sivas, Turkey. Critical revision of the manuscript.

**Keywords:** Nigella sativa, Vitis vinifera, cytokine, inflammation, wound healing

## Abstract

**Purpose:**

This study aimed to experimentally investigate the effects of *Nigella sativa* and *Vitis vinifera* oils on intraoral wound healing in rats through biochemical, histopathological, and histomorphometric evaluations.

**Methods and Materials:**

Sixty male Wistar albino rats (4–6 months old) were divided into four groups: Control (C, n = 6), Saline (S, n = 18), *Nigella sativa* (Ns, n = 18), and *Vitis vinifera* (Vv, n = 18). A full-thickness circular wound of 3.5 mm was created in the palate. Treatments were administered daily via oral gavage until the day of sacrifice. Serum IL-1β, IL-6, and IL-10 levels were measured from collected blood samples using the enzyme-linked immunosorbent assay (ELISA) method. Full-thickness tissue samples (1 × 1 cm, including healthy tissue) underwent routine histological processing. The resulting intraoral photographs were used for digital wound area measurements.

**Results:**

In the experimental groups, cytokine levels in blood samples collected during the first week differed significantly compared with the control group. Significant differences in IL-1β, IL-6, and IL-10 levels were observed among the subgroups on days 7, 14, and 21 (*p *< 0.05). Histological evaluation showed a significant reduction in intraepithelial ulceration in the Ns and Vv groups (*p* < 0.05), along with increased re-epithelialisation. Wound area measurements revealed significant differences between the Ns and Vv groups and the S group (*p* < 0.05).

**Conclusion:**

*Nigella sativa* and *Vitis vinifera* support intraoral wound healing through modulation of pro- and anti-inflammatory cytokines. To the best of our knowledge, this study represents the first experimental comparison of these two phytochemicals in an intraoral wound healing model.

A wound is defined as a condition resulting in functional loss due to disruption of tissue integrity caused by environmental factors or therapeutic interventions.^16^ Wound healing is a complex and organised process divided into interconnected phases: hemostasis, inflammation, proliferation, and remodelling.^15^ Communication among various variables within this process is supported by the expression of cytokines such as interleukins (IL), tumour necrosis factor-alpha (TNF-α), and chemokines, along with adhesive molecules and growth factors including platelet-derived growth factor (PDGF), platelet-activating factor (PAF), and fibroblast growth factor-2 (FGF-2).^11^


Intraoral or perioral tissue damage resulting from thermal, chemical, or electrical burns heals by secondary intention through wound contraction and epithelialisation.^20^ In addition, wound healing in many surgical procedures performed in dentistry occurs through secondary healing.^5^ Developing strategies to minimise postoperative wound-healing complications in oral surgery is currently a major focus of clinical research in modern dentistry.^56^ Researchers continue to investigate novel formulations and herbal compositions to develop low-cost, effective, and sustainable treatment methods for wound management and therapy.^43^ The oral cavity hosts approximately 700 species of microorganisms, including bacteria, fungi, viruses, and protozoa.^54^ The proliferation of pathogenic microorganisms in oral surgery can lead to postoperative inflammation, infection, and wound dehiscence.^6^ Therefore, the use of antiseptic agents can help limit complications and contribute to the healing process.^42^


Scientific research has highlighted the significant effects of medicinal plants and phytochemicals on human tissues and their positive potential in tissue repair and wound healing.^7^ A literature review of the past decade on the use of medicinal plants in wound healing, both *in vitro* and *in vivo*, confirmed that 109 genera from 62 plant families have been utilised in clinical studies. The data demonstrated that these plants significantly enhance healing through the presence of secondary metabolites – such as flavonoids, alkaloids, saponins, tannins, terpenoids, and phenolic compounds – that exhibit anti-inflammatory, antimicrobial, and antioxidant properties via multiple mechanisms at different stages of healing. These findings indicate that medicinal plants may provide viable alternatives to conventional treatments.^9^
*Nigella sativa* (Ns)^18^ and *Vitis vinifera* (Vv)^39^ are medicinal plants that act as antibacterial, antioxidant, and anti-inflammatory agents. NS belongs to the *Nigella* genus of the *Ranunculaceae* family.^40^ The bioactive compound thymoquinone (TQ) contained in Ns has been evaluated in various studies, revealing antimicrobial, anti-inflammatory, antiallergic, antioxidant, antineoplastic, and antidiabetic properties.^45^ Numerous studies have also examined plant materials derived from Vv, a grapevine species. Its proanthocyanidin content is suggested to possess strong immunomodulatory properties. Grape seed extract exhibits several biological activities, including anti-inflammatory, anticarcinogenic, and antioxidant effects.^12^


The wound-healing properties of Ns^50^ and Vv^12^ have been investigated in cutaneous models, and Tabaru and Rüzgar^50^ explored the effects of Ns on oral wound healing. However, no comparative study has yet examined the effects of Ns and Vv on intraoral mucosal wounds in the field of oral surgery. Given the unique healing dynamics of the oral mucosa, the application of these compounds within the oral cavity is of foremost importance for developing new therapeutic strategies in oral surgery.

The present study aimed to evaluate the effects of Ns and Vv on intraoral wound healing in a rat palatal mucosal defect model and to determine their potential as adjunctive treatments in oral surgical wound management by assessing biochemical (IL-1β, IL-6, IL-10), histopathological, and histomorphometric outcomes.

## Methods and Materials

This experimental study was conducted to evaluate the effects of *Nigella sativa* and *Vitis vinifera* on intraoral wound healing in a rat model. The study was approved by the Adıyaman University Animal Experiments Ethics Committee (protocol number: 2024/018) and conducted in accordance with international guidelines for animal research.

### Animals

A total of 60 adult male Wistar albino rats (300–350 g, 4–6 months old) were used. (They were obtained from the Adıyaman University Experimental Animals Production, Application, and Research Centre.) The animals were housed under standard laboratory conditions (22 ± 2°C, 12-h light/dark cycle) with ad libitum access to food and water. All rats were acclimatised to the environment one week before the experiment. The experimental procedures were performed at the Adıyaman University Experimental Animals Production, Application, and Research Centre.

### Wound Model

After general anaesthesia was achieved with intramuscular administration of 30 mg/kg ketamine-HCl (Ketalar, Eczacıbaşı) and 5 mg/kg xylazine-HCl (Rompun, Bayer), a circular full-thickness mucosal defect measuring 3.5 mm in diameter was created in the palatal region. After haemostasis was ensured, the wound was left to heal by secondary intention. The day of defect creation was recorded as day 0 for all groups. The defect size and location were standardised among all animals to ensure consistency. Postoperatively, all rats were maintained under identical feeding conditions. For analgesia, Carprofen 4 mg/kg (Rimadyl, Pfizer) was administered subcutaneously once daily for three days. Routine care and body-weight monitoring were performed throughout the experiment.

### Experimental Groups and Treatments

The rats were randomly divided into one control group (n = 6) and three experimental groups (n = 18 each): saline, Ns, and Vv. Each group was further divided into three subgroups (n = 6) according to sacrifice days (7, 14, and 21). The Ns and Vv extracts (100% cold-pressed black seed oil and 100% cold-pressed grape seed oil, free from chemical or thermal processing; Saraçoğlu Tourism and Environmental Technologies Industry and Trade, Turkey) were administered daily by gavage (1 mL/kg) until the day of sacrifice. The saline group received only physiological saline (0.9%) without any additional treatment. All procedures were performed under aseptic conditions. This dosage was selected based on established preclinical studies demonstrating safety and efficacy in wound healing models at 0.8–2 ml/kg/day.^3,21,23,25,31,47
^


### Sacrifice Protocol and Sample Collection

Animals in the control group were sacrificed on day 0 for standardisation purposes. According to the experimental protocol, animals from the remaining groups were sacrificed on days 7, 14, and 21. Prior to sacrifice, all rats were anaesthetised, and intracardiac blood samples were collected. The samples were centrifuged at 3000 g for 10 minutes to obtain serum for biochemical analysis and stored at −80 °C until testing. Subsequently, intraoral tissue samples containing the secondary wound area and adjacent healthy tissue (1 × 1 cm) were collected and placed in biopsy containers with 10% buffered formalin.

### Biochemical Analysis

Blood samples were collected immediately before sacrifice via cardiac puncture. Serum IL-1β, IL-6, and IL-10 levels were measured using enzyme-linked immunosorbent assay (ELISA) kits (YLbiont, Shanghai YL Biotech), according to the manufacturer’s instructions. All parameters were analysed using a BioTek ELx800 ELISA reader.

### Histopathological and Histomorphometric Analysis

The tissue samples were fixed in 10% neutral buffered formalin, processed using routine histological techniques, and embedded in paraffin. Sections (5 µm thick) were stained with haematoxylin–eosin (H&E), Masson’s trichrome, Verhoeff, and toluidine blue for histopathological evaluation. The parameters assessed included intraepithelial ulceration, re-epithelialisation, fibroblast proliferation, inflammation, vascularisation, connective tissue oedema, fibrosis, and mast cell density. Samples were scored by a blinded pathologist according to predefined criteria (0 = none, 1 = mild, 2 = moderate, 3 = severe).^23^ Wound surface areas were photographed at a fixed distance and magnification using a 5 MP 500× digital LCD stereomicroscope mounted on a custom-made stand to ensure constant positioning. The wound area measurements were performed using ImageJ software (ImageJ and NIH Image Software, National Institutes of Health, Bethesda, MD).

### Statistical Analysis

All data were analysed using the SPSS 28.0 software package. Normality was assessed with the Shapiro–Wilk and Kolmogorov–Smirnov tests. Intergroup comparisons of biochemical and histopathological data were conducted using Mann–Whitney U tests. Wound surface area measurements were compared using paired-sample t-tests. Results are presented as mean ± standard deviation (SD). A *p* value < 0.05 was considered statistically significant.

## Results

### Biochemical Findings

Serum levels of IL-1β, IL-6, and IL-10 were measured in the control (baseline, day 0), saline, Ns and Vv groups on days 7, 14, and 21 using the ELISA method. The differences in cytokine levels between the control group (day 0) and the experimental groups (day 7) were statistically significant (*p* < 0.05). The only exception was the Ns group, in which the difference in IL-10 levels was not significant (*p* = 0.345) (Table 1).

**Table 1 Table1:** Comparison of cytokine levels between the control group (day 0) and experimental groups (day 7)

	Control (c) n = 6	Saline (S) n = 6	*Nigella sativa *(Ns) n = 6	*Vitis vinifera *(Vv) n = 6	*p* value
IL-6 (ng/L)	2.50 ±0.13	12.44 ± 4.40	5.60 ± 1.85	3.65 ± 0.95	C-S = 0.028* C-Ns = 0.028* C-Vv = 0.028*
IL-10 (pg/ml)	20.68 ± 6.22	9.55 ± 3.70	17.16 ± 2.12	12.09 ± 2.82	C-S = 0.028* C-Ns = 0.345 C-Vv = 0.046*
IL-1β (pg/L)	561.56 ± 41.70	991.18 ± 155.23	865.95 ± 115.81	873.55 ± 253.37	C-S = 0.028* C-Ns = 0.028* C-Vv = 0.028*
n: number of animals; **p* < 0.05: indicates a statistically significant difference between groups (Mann–Whitney U test).					

Significant differences were also observed among the subgroups on days 7, 14, and 21 (*p* < 0.05) (Table 2). On day 7, the Ns and Vv groups exhibited significantly lower IL-6 levels compared to the saline group, with the Vv group showing even lower levels than the Ns group (*p* = 0.028). The Ns group demonstrated higher IL-10 levels (*p* < 0.05). IL-1β levels did not differ significantly among the groups on day 7 (*p* > 0.05).

**Table 2 Table2:** Comparison of cytokine levels among the experimental groups

Cytokine	Day	Saline (S) n = 6	*Nigella sativa* (Ns) n = 6	*Vitis vinifera* (Vv) n = 6	*p* value
**IL-6 (ng/L)**	7	12.44 ± 4.40	5.60 ± 1.85	3.65 ± 0.95	Ns-S = 0.028*; Vv-S = 0.028*; Vv-Ns = 0.028*
	14	3.28 ± 0.23	3.44 ± 0.46	2.94 ± 0.43	Ns-S = 0.345; Vv-S = 0.046*; Vv-Ns = 0.028*
	21	2.95 ± 0.30	2.84 ± 0.15	2.61 ± 0.20	Ns-S = 0.600; Vv-S = 0.028*; Vv-Ns = 0.028*
**IL-10 (pg/ml)**	7	9.55 ± 3.70	17.16 ± 2.12	12.09 ± 2.82	Ns-S = 0.028*; Vv-S = 0.116; Vv-Ns = 0.028*
	14	4.04 ± 3.16	21.48 ± 1.03	13.20 ± 2.46	Ns-S = 0.028*; Vv-S = 0.028*; Vv-Ns = 0.028*
	21	19.98 ± 4.0	16.54 ± 5.18	15.19 ± 6.51	Ns-S = 0.116; Vv-S = 0.116; Vv-Ns = 0.600
**IL-1β (pg/L)**	7	991.18 ± 155.23	865.95 ± 115.81	873.55 ± 253.37	Ns-S = 0.116; Vv-S = 0.345; Vv-Ns = 0.917
	14	673.5 ± 99.7	742.2 ± 55.8	657.6 ± 101.0	Ns-S = 0.028*; Vv-S = 0.463; Vv-Ns = 0.046*
	21	649.3 ± 59.1	572.6 ± 80.3	586.1 ± 36.8	Ns-S = 0.075; Vv-S = 0.046*; Vv-Ns = 0.753
n: number of animals; **p* < 0.05: indicates a statistically significant difference between groups (Mann–Whitney U test).					

On day 14, IL-6 levels in the Vv group were significantly lower than those in the saline and Ns groups (*p* = 0.046 and *p* = 0.028, respectively). IL-1β levels were increased in the Ns group compared to both the saline and Vv groups (*p* = 0.028 and *p* = 0.046, respectively). IL-10 levels were significantly higher in both the Ns and Vv groups compared to saline (*p* = 0.028), and the difference between Ns and Vv was also statistically significant.

On day 21, IL-6 levels in the Vv group remained significantly lower than those in the saline and Ns groups (*p* = 0.028), while IL-1β levels in the Vv group were lower compared to the saline group (*p* = 0.046). No significant differences in IL-10 levels were observed among the groups on day 21 (*p* > 0.05).

### Histopathological and Histomorphometric Findings

Histopathological parameters including intraepithelial ulceration, re-epithelialisation, fibroblast proliferation, inflammation, vascularisation, connective tissue oedema, fibrosis, and mast cell density were evaluated in the saline, Ns, and Vv groups on days 7, 14, and 21. All specimens were examined microscopically by the same histologist under blinded conditions (Fig 1). Significant differences were observed both among the groups and across time points (Tables 3a and 3b).

**Fig 1a to d Fig1atod:**
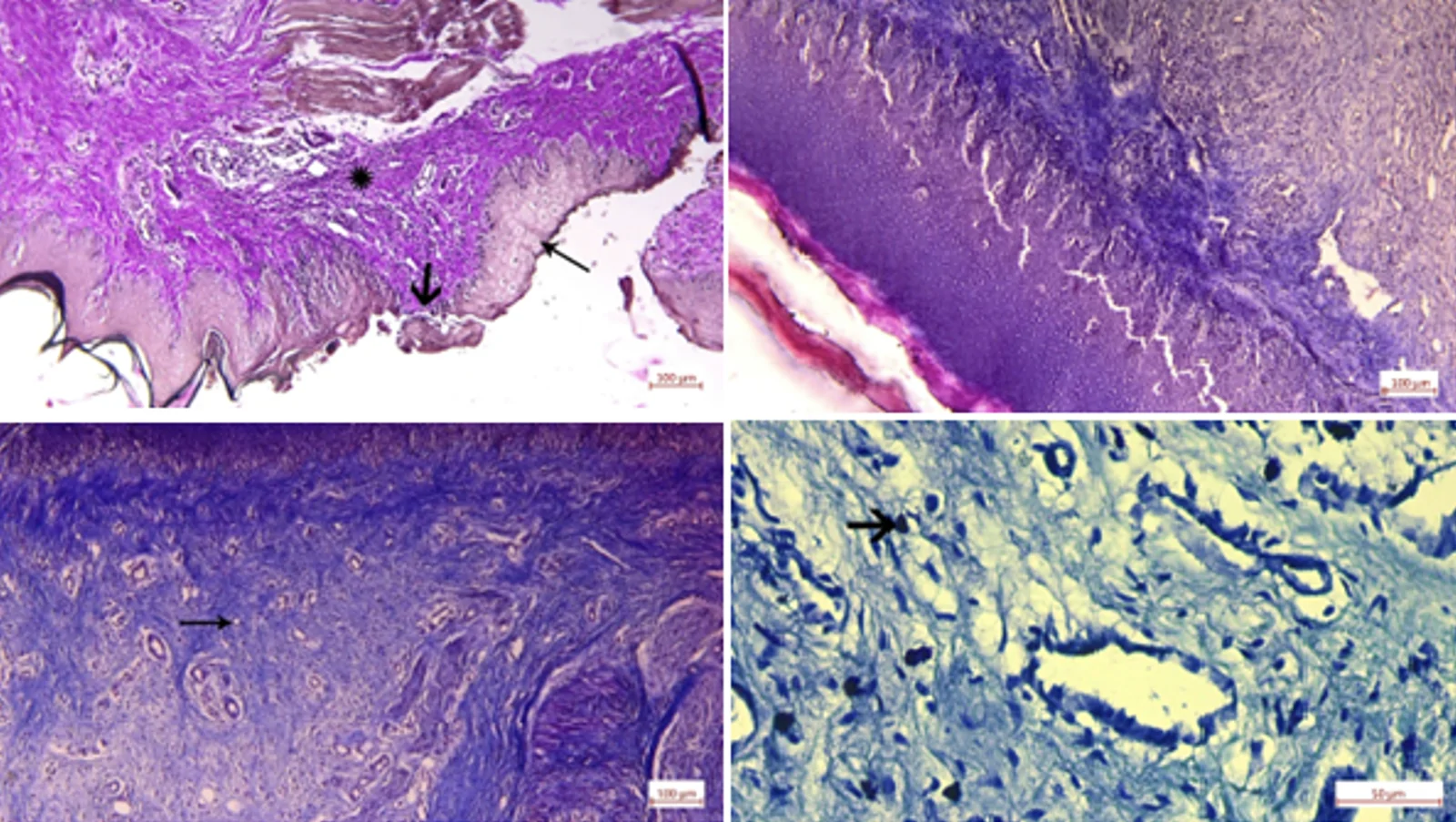
Histological sections of the experimental groups. (a) Palatal mucosal epithelium and connective tissue area from the saline group on day 7, stained with H&E (×10 objective magnification). Thick arrow: epithelial ulcer; star: inflammation; thin arrow: squamous intraepithelial lesion. (b) Connective tissue area from the Nigella sativa group on day 7, stained with Masson’s trichrome (×10 objective magnification). Finding: fibrosis. (c) Connective tissue area from the Vitis vinifera group on day 7, stained with Masson’s trichrome (×10 objective magnification). Arrow: blood vessel. (d) Connective tissue area from the saline group on day 21, stained with toluidine blue (×40 objective magnification). Arrow: Mast cell.

**Table 3a Table3a:** Histopathological evaluation of wound healing parameters

Parameter	Day	Saline (S) n = 6	*Nigella sativa *(Ns) n = 6	*Vitis vinifera* (Vv) n = 6	*p* value
**Intraepithelial ulceration**	7	2.50 ± 1.21	0.67 ± 0.75	0.17 ± 0.84	Ns-S = 0.002*;Vv-S = 0.005*; Vv-Ns = 0.203
	14	0.50 ± 0.82	0.66 ± 0.40	0.17 ± 0.82	Ns-S = 0.363; Vv-S = 0.363; Vv-Ns = 0.363
	21	0.83 ± 1.03	0.50 ± 0.75	0.67 ± 0.75	Ns-S = 0.465; Vv-S = 0.611; Vv-Ns = 0.611
**Re-epithelialisation**	7	0.50 ± 0.75	2.33 ± 1.17	2.67 ± 0.52	Ns-S = 0.012*;Vv-S = 0.001*;Vv-Ns = 0.175
	14	2.33 ± 0.75	2.50 ± 0.75	2.83 ± 0.82	Ns-S = 0.611;Vv-S = 0.076; Vv-Ns = 0.363
	21	2.00 ± 1.03	2.67 ± 1.03	2.67 ± 0.40	Ns-S = 0.175;Vv-S = 0.175; Vv-Ns = 0.363
**Fibroblast proliferation**	7	2.33 ± 0.52	2.67 ± 0.52	2.50 ± 0.98	Ns-S = 0.175;Vv-S = 0.741; Vv-Ns = 0.695
	14	2.67 ± 0.89	2.67 ± 0.89	2.83 ± 0.75	Ns-S = 1.000;Vv-S = 0.611; Vv-Ns = 0.611
	21	2.50 ± 1.21	1.83 ± 0.84	1.33 ± 0.75	Ns-S = 0.235;Vv-S = 0.013*; Vv-Ns = 0.203
**Inflammation**	7	2.50 ± 0.63	2.50 ± 0.63	2.17 ± 0.82	Ns-S = 1.000;Vv-S = 0.363; Vv-Ns = 0.363
	14	2.83 ± 0.55	2.33 ± 0.55	2.17 ± 0.41	Ns-S = 0.076;Vv-S = 0.025*; Vv-Ns = 0.363
	21	2.17 ± 0.55	1.67 ± 0.55	1.17 ± 0.55	Ns-S = 0.076;Vv-S = 0.012*; Vv-Ns = 0.076
n: number of animals; * *p* < 0.05: indicates a statistically significant difference between groups (Mann–Whitney U test).					

**Table 3b Table3b:** Histopathological evaluation of connective tissue healing parameters

Parameter	Day	Saline (S) n = 6	*Nigella sativa *(Ns) n = 6	*Vitis vinifera *(Vv) n = 6	*p* value
**Vascularisation**	7	1.17 ± 1.05	1.83 ± 1.03	2.67 ± 0.41	Ns-S = 0.175; Vv-S = 0.017*; Vv-Ns = 0.004*
	14	2.17 ± 0.75	2.00 ± 0.75	2.83 ± 0.41	Ns-S = 0.611; Vv-S = 0.102; Vv-Ns = 0.004*
	21	2.17 ± 0.82	2.50 ± 0.82	2.67 ± 0.75	Ns-S = 0.363; Vv-S = 0.213; Vv-Ns = 0.611
**Connective tissue oedema**	7	2.17 ± 1.05	2.67 ± 1.05	2.50 ± 0.75	Ns-S = 0.296; Vv-S = 0.465; Vv-Ns = 0.611
	14	2.67 ± 0.41	2.50 ± 0.41	2.33 ± 0.75	Ns-S = 0.363; Vv-S = 0.363; Vv-Ns = 0.611
	21	2.50 ± 0.52	2.17 ± 0.52	1.33 ± 0.98	Ns-S = 0.175; Vv-S = 0.013*; Vv-Ns = 0.093
**Fibrosis**	7	2.00 ± 0.89	3.00 ± 0.89	2.50 ± 0.55	Ns-S = 0.041*; Vv-S = 0.415; Vv-Ns = 0.076
	14	3.00 ± 0.52	2.67 ± 0.52	2.17 ± 0.84	Ns-S = 0.175; Vv-S = 0.004*; Vv-Ns = 0.203
	21	2.17 ± 0.75	2.33 ± 0.75	1.50 ± 0.41	Ns-S = 0.611; Vv-S = 0.025*; Vv-Ns = 0.004*
**Mast cell density**	7	2.67 ± 0.55	2.50 ± 0.55	2.00 ± 0.55	Ns-S = 0.510; Vv-S = 0.030*; Vv-Ns = 0.076
	14	2.67 ± 0.55	2.17 ± 0.55	2.00 ± 0.41	Ns-S = 0.076; Vv-S = 0.030*; Vv-Ns = 0.363
	21	2.67 ± 0.52	1.67 ± 0.52	0.67 ± 0.52	Ns-S = 0.008*; Vv-S < 0.001*; Vv-Ns = 0.008*
n: number of animals * *p* < 0.05: indicates a statistically significant difference between groups (Mann–Whitney U test).					

On day 7, intraepithelial ulceration scores were significantly lower in the Ns and Vv groups compared with the saline group (*p* = 0.002 and *p* = 0.005, respectively). Re-epithelialisation was significantly more advanced in both Ns and Vv than in saline (*p* = 0.012 and *p *= 0.001, respectively). Fibrosis scores were higher in the Ns group compared with saline (*p *= 0.041), while mast cell density was lower in Vv than in saline (*p* = 0.030). No significant differences were observed in fibroblast proliferation, inflammation, vascularisation, or connective tissue oedema at this point.

On day 14, inflammation scores were significantly lower in Vv compared with saline (*p* = 0.025), and fibrosis scores were also lower in Vv than in saline (*p* = 0.004). Vascularisation was higher in Vv compared with NS (*p* = 0.004), and mast cell density was lower in Vv than in saline (*p* = 0.030). No significant differences were detected in ulceration or re-epithelialisation among the groups.

On day 21, fibroblast proliferation was significantly lower in Vv compared with saline (*p* = 0.013). Inflammation scores were lower in both Ns and Vv compared with saline (*p* = 0.076 and *p* = 0.012, respectively). Connective tissue oedema was reduced in Vv compared with saline (*p* = 0.013). Fibrosis scores were significantly lower in Vv compared with both saline and Ns (*p* = 0.025 and *p* = 0.004, respectively). Mast cell density in Vv was significantly reduced compared with both saline and Ns (*p* < 0.001 and *p* = 0.008, respectively). Complete re-epithelialisation was observed in the Ns and Vv groups on day 21, whereas it was absent in the saline group.

Intraoral macroscopic images of the saline, Ns and Vv groups were evaluated on days 7, 14, and 21. Visual analysis demonstrated progressive wound closure over time in all groups (Fig 2). Statistically significant differences were observed in wound size measurements among the groups on days 7, 14, and 21 (Table 4).

**Fig 2 Fig2:**
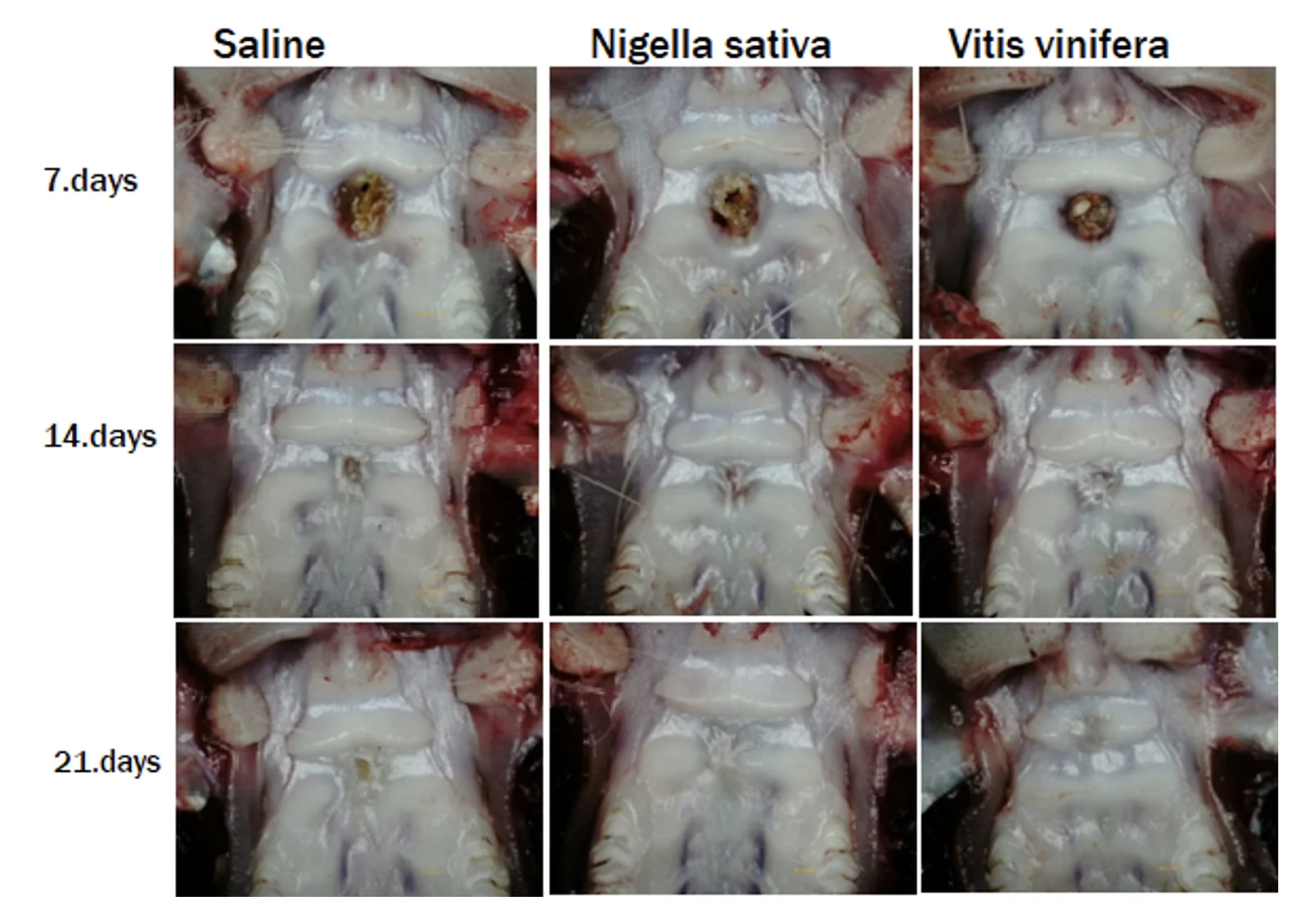
Representative intraoral macroscopic images of the experimental groups. *Images were taken under standardized conditions (5 MP digital stereomicroscope, fixed distance).

**Table 4 Table4:** Comparison of wound surface areas among experimental groups

Day	Control (C) (mm^2^) n = 6	Saline (S) (mm^2^) n = 6	*Nigella sativa* (Ns) (mm^2^) n = 6	*Vitis vinifera* (Vv) (mm^2^) n = 6	*p* value
0	9.40	–	–	–	–
7	–	8.45 ± 0.68	5.26 ± 0.68	4.54 ± 0.49	Ns-S < 0.001*; Vv-S < 0.001*;Vv-Ns = 0.020*
14	–	1.83 ± 0.17	1.31 ± 0.17	1.12 ± 0.19	Ns-S = 0.001*; Vv-S < 0.001*;Vv-Ns = 0.043*
21	–	0.78 ± 0.14	0.44 ± 0.14	0.28 ± 0.07	Ns-S = 0.002*; Vv-S < 0.001*; Vv-Ns = 0.013*
n: number of animals **p* < 0.05: indicates a statistically significant difference between groups (paired samples test).					

On day 7, the Ns and Vv groups exhibited visibly smaller wound areas compared with the saline group. On day 14, wound contraction was more pronounced in both the Ns and Vv groups, with the Vv group showing the greatest reduction in wound size. By day 21, nearly complete wound closure was observed in the Ns and Vv groups, whereas wounds in the saline group had not yet fully closed (Fig 3).

**Fig 3 Fig3:**
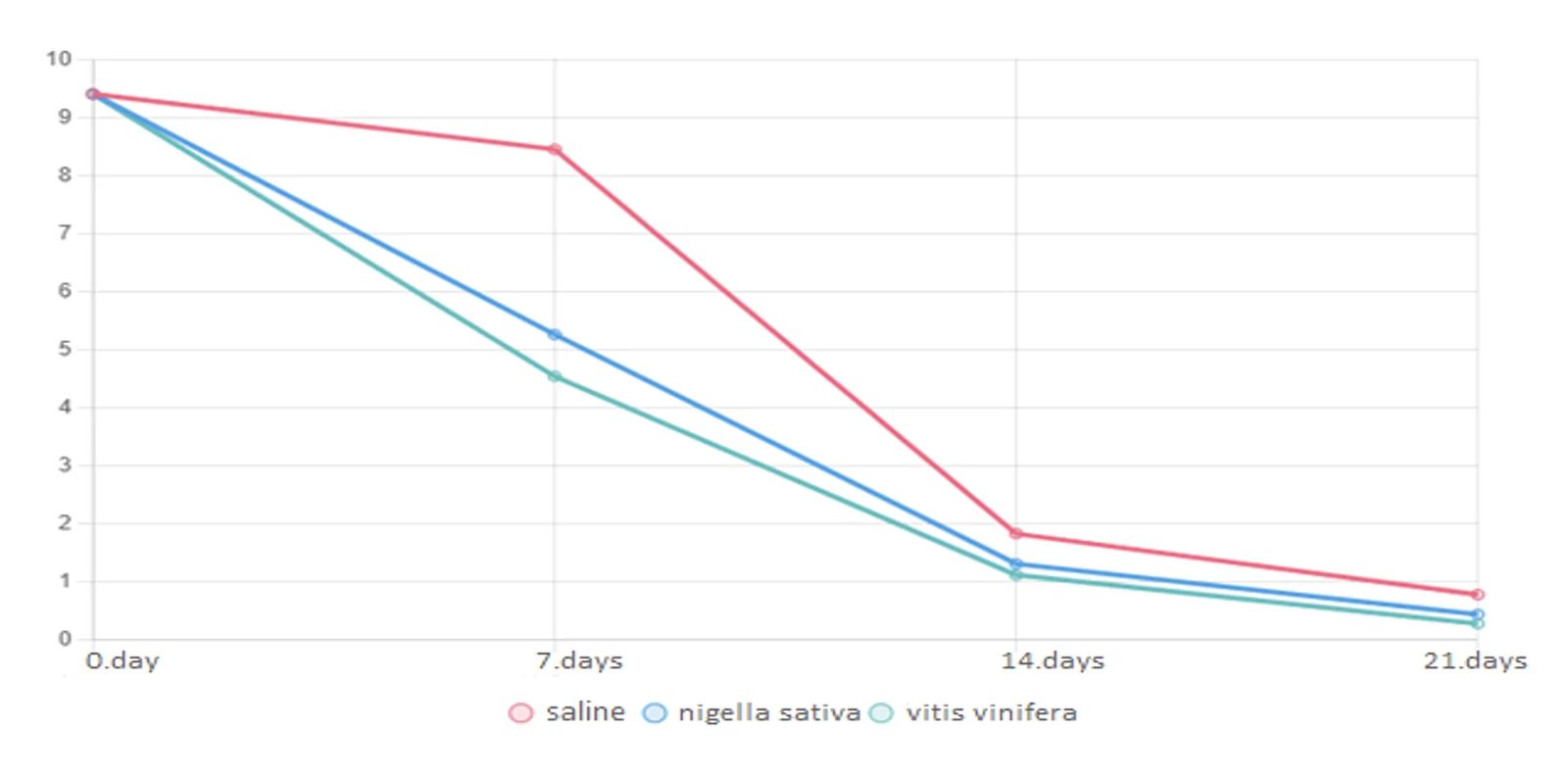
Time-dependent changes in wound surface area.

## Discussion

Wound healing is a complex process in which the body orchestrates a series of coordinated events to maintain homeostasis. The longer the healing process takes, the greater the risk of harmful substances and microbial products entering the body and causing damage.^10^ Consequently, secondary healing takes more time and is more prone to microbial contamination.^27^ In oral surgery, wound healing occurs within the oral cavity and is influenced by various factors such as the heterogeneous oral flora, mechanical trauma from mastication, and increased dental plaque accumulation due to poor oral hygiene, which collectively delay the healing process. Such delays may increase the risk of infection and bacteraemia.^29^ Infections and inflammation of the oral mucosa can lead to systemic inflammation and sepsis through bacteraemia.^51^ Therefore, preventing oral mucosal infections and delayed wound healing is significant. Studies have demonstrated that nonsteroidal anti-inflammatory drugs (NSAIDs) positively affect the wound-healing process following oral surgeries.^1,41,46
^ Accordingly, phytochemicals known for their anti-inflammatory properties have been increasingly investigated for their potential effects on wound healing.^2,8,19,22,50
^


One of the phytochemicals studied is *Nigella sativa*. The main bioactive compound of Ns, thymoquinone (TQ), exerts its anti-inflammatory effects by reducing the expression of proinflammatory cytokines (IL-1β, IL-6, TNF-α) through inhibition of the nuclear factor kappa B (NF-κB) signalling pathway.^27^ Mehri et al^32^ demonstrated that Ns possesses anti-inflammatory properties and positively influences various stages of wound healing.

During the wound-healing process, proinflammatory cytokines such as IL-1β, IL-6, and TNF-α play key roles in the inflammatory phase, strongly upregulating the healing process.^14^ However, anti-inflammatory cytokines also exert significant effects during wound healing. For instance, IL-10, an anti-inflammatory cytokine, plays an essential role in limiting or terminating inflammatory responses and contributes to the growth and differentiation of keratinocytes and endothelial cells.^34^ The balance between these pro- and anti-inflammatory responses is critical for proper healing. Disruption of this balance may result in excessive tissue destruction, scarring, infection, or delayed healing.^28^


Experimental studies by Hijazy et al^22^ and Umar et al^53^ showed that oral-gavage-administered thymoquinone reduced proinflammatory mediators while increasing IL-10 levels in different inflammatory models. However, a reduction in IL-6 levels and an increase in IL-10 levels were both statistically significant compared with saline. On day 14, IL-1β levels were higher in the Ns group than in saline. Haq et al^19^ associated this finding with the modulatory effects of Ns on macrophage activity. Histopathologically, the results are consistent with the findings of Mohammed et al^33^ and Selçuk et al,^48^ who reported a decrease in ulceration and an increase in re-epithelialisation in the groups treated with *Nigella sativa*. Ulceration scores were lower and re-epithelialisation scores were significantly higher in the *Nigella sativa* group compared to the saline group. Despite the contradictory findings regarding vascularisation among studies, our results indicated no significant difference in vascularisation between the Ns and saline groups.^26,38
^


Another phytochemical investigation is *Vitis vinifera*. Negro et al^37^ confirmed the anti-inflammatory effects of grape seed extract through its reduction of inflammatory mediators. The proanthocyanidin content of Vv is known for its potent antioxidant and anti-inflammatory properties. Proanthocyanidins neutralise reactive oxygen species (ROS), thereby reducing oxidative stress and helping to control inflammation at the wound site.^31^ Another key compound, resveratrol, exhibits anti-inflammatory activity by targeting nuclear factor kappa B (NF-κB), suppressing the expression of proinflammatory cytokines, and regulating genes involved in eicosanoid synthesis.^30,44
^ Zheng et al^55^ and Al-Warhi et al^4^ reported that *Vitis vinifera* extracts reduced IL-1β and IL-6 levels and promoted vascularisation and epithelial regeneration in different wound models. In our study, Vv groups showed significantly lower IL-1β levels than saline groups on day 21. IL-6 levels decreased, and IL-10 levels increased significantly on day 14.

Niknami et al^36^ reported that Vv seed extract and oil significantly reduced the ulceration index, while Nayak et al^35^ showed faster epithelialisation. Al-Warhi et al^4^ also demonstrated that Vv strongly stimulated vascularisation in a rabbit wound model. Similarly, in our study, ulceration was reduced in the Vv group, and both re-epithelialisation and vascularisation were higher compared to the saline group.

The oral cavity harbours a complex microbial ecosystem that plays a critical role in both oral health and wound healing. While the resident microbiota contributes to tissue homeostasis, excessive colonisation by pathogenic microorganisms may impair healing by prolonging inflammation, increasing tissue destruction, and promoting local infection.^51^ Therefore, microbial control is considered an important determinant of successful oral wound healing. Oral infections and excessive bacterial colonisation may delay healing and prolong inflammatory responses. Previous studies have emphasised the relationship between oral microbial burden and tissue healing and highlighted the importance of maintaining microbial balance during the repair process.^17,49
^ In addition to their anti-inflammatory and antioxidant properties, both *Nigella sativa* and *Vitis vinifera* possess well-documented antimicrobial activities. Thymoquinone, the principal bioactive constituent of *Nigella sativa*, has been shown to exhibit broad-spectrum antibacterial effects against a variety of pathogenic microorganisms and to inhibit microbial growth and colonisation. Likewise, *Vitis vinifera* contains biologically active polyphenols, particularly resveratrol and proanthocyanidins, which have demonstrated antimicrobial activity through the inhibition of bacterial adhesion, biofilm formation, and microbial proliferation.^9,18,31
^ These antimicrobial properties may have contributed to the favourable healing outcomes observed in the present study by reducing microbial burden at the wound site and creating a more conducive environment for tissue repair and regeneration.

Our findings demonstrate that *Nigella sativa* and *Vitis vinifera* promote healing in a rat palatal mucosal wound model. To the best of our knowledge, this is the first experimental study directly comparing the effects of these two phytochemicals on intraoral wound healing. The more pronounced effect of *Vitis vinifera* on wound contraction and reduction of proinflammatory cytokines may be related to its higher proanthocyanidin content, whereas the greater increase in IL-10 observed with *Nigella sativa* likely reflects the immunomodulatory effects of thymoquinone reported previously.^33^ The differences observed between the two agents may be attributed to variations in the chemical composition and biological activity of their bioactive components. These findings suggest that *Nigella sativa* and *Vitis vinifera* may represent promising, cost-effective natural adjuncts for the management of oral wounds.

Although our study provided important findings on the effects of Ns and Vv on intraoral wound healing, several limitations should be considered. First, the study was conducted exclusively on male Wistar albino rats. The impact of sex on wound healing may differ due to hormonal factors.^13^ Including female rats would have improved the generalisability of the results. Second, the study focused solely on the palatal mucosa model, so the effects on other oral regions remain unknown. Third, dosage optimisation was not performed, and the applied doses (1 ml/kg) may not directly translate to human application. Testing a broader dose range would better clarify the therapeutic potential of both extracts. Lastly, the study was limited to a 21-day observation period. Long-term follow-up studies examining remodelling and sustained effects at the wound site would be beneficial to assess the durability of Ns and Vv’s effects.

## Conclusion

Based on biochemical, histopathological, and stereomicroscopic findings, the application of *Nigella sativa* and *Vitis vinifera* supports previous studies demonstrating their positive effects on wound healing. These findings suggest that *Vitis vinifera* and *Nigella sativa* can be used as effective complementary therapies in oral surgery and the treatment of intraoral ulcers. *Vitis vinifera* demonstrated faster wound closure, lower inflammatory cytokine levels, and increased vascularisation compared to *Nigella sativa*. Further research is needed to confirm their clinical applicability. While both *Nigella sativa* and *Vitis vinifera* exhibit anti-inflammatory properties and promote wound healing, additional studies are required to identify the specific active agents and elucidate their underlying mechanisms of action.

### Acknowledgements

The authors thank the laboratory team and veterinarians.

#### Data availability

The data sets used and/or analysed during the current study are available from the corresponding author on reasonable request.

#### Funding

This study was supported by Adıyaman University Scientıfic Research Projects Coordination, Project No: DHFDUP/2024-0002.

#### Ethics declarations

The study was approved by the Adıyaman University Animal Experiments Ethics Committee (protocol number: 2024/018).

#### Conflict of interest

The authors declare that there is no conflict of interest.

## References
